# Impact of rapid molecular testing on diagnosis, treatment and management of community-acquired pneumonia in Norway: a pragmatic randomised controlled trial (CAPNOR)

**DOI:** 10.1186/s13063-022-06467-7

**Published:** 2022-08-01

**Authors:** Sondre Serigstad, Christian Ritz, Daniel Faurholt-Jepsen, Dagfinn Markussen, Marit H. Ebbesen, Øyvind Kommedal, Rune O. Bjørneklett, Lars Heggelund, Tristan W. Clark, Cornelis H. van Werkhoven, Siri T. Knoop, Elling Ulvestad, Harleen M. S. Grewal, The CAPNOR study group, R. Bjørneklett, T. W. Clark, M. Ebbesen, D. Faurholt-Jepsen, H. M. S. Grewal, L. Heggelund, S. T. Knoop, Ø. Kommedal, D. Markussen, P. Ravn, C. Ritz, S. Serigstad, E. Ulvestad, C. H. Van Werkhoven

**Affiliations:** 1 Emergency Care Clinic, Haukeland University Hospital, Bergen, Norway; 2 Department of Clinical Medicine, Faculty of Medicine, University of Bergen, Bergen, Norway; 3 Department of Clinical Science, Bergen Integrated Diagnostic Stewardship cluster, Faculty of Medicine, University of Bergen, Bergen, Norway; 4 National Institute of Public Health, University of Southern Denmark, Copenhagen, Denmark; 5 Department of Infectious Diseases, Rigshospitalet, Copenhagen, Denmark; 6 Department of Microbiology, Haukeland University Hospital, Bergen, Norway; 7 Department of Internal Medicine, Vestre Viken Hospital Trust, Drammen, Norway; 8 School of Clinical and Experimental Sciences, Faculty of Medicine, University of Southampton, Southampton, UK; 9 Julius Center for Health Sciences and Primary Care, University Medical Center Utrecht, Utrecht, The Netherlands

**Keywords:** Community-acquired pneumonia, Respiratory tract infections, Microbiological testing, Rapid diagnostics, FilmArray Pneumonia Panel, Syndromic PCR panel, Molecular testing, Induced sputum, Antimicrobial treatment

## Abstract

**Background:**

Community-acquired pneumonia (CAP) causes a large burden of disease. Due to difficulties in obtaining representative respiratory samples and insensitive standard microbiological methods, the microbiological aetiology of CAP is difficult to ascertain. With a few exceptions, standard-of-care diagnostics are too slow to influence initial decisions on antimicrobial therapy. The management of CAP is therefore largely based on empirical treatment guidelines. Empiric antimicrobial therapy is often initiated in the primary care setting, affecting diagnostic tests based on conventional bacterial culture in hospitalized patients. Implementing rapid molecular testing may improve both the proportion of positive tests and the time it takes to obtain test results. Both measures are important for initiation of pathogen-targeted antibiotics, involving rapid de-escalation or escalation of treatment, which may improve antimicrobial stewardship and potentially patient outcome.

**Methods:**

Patients presenting to the emergency department of Haukeland University Hospital (HUH) in Bergen, Norway, will be screened for inclusion into a pragmatic randomised controlled trial (RCT). Eligible patients with a suspicion of CAP will be included and randomised to receive either standard-of-care methods (standard microbiological testing) or standard-of-care methods in addition to testing by the rapid and comprehensive real-time multiplex PCR panel, the BioFire® FilmArray® Pneumonia Panel *plus* (FAP *plus*) (bioMérieux S.A., Marcy-l’Etoile, France). The results of the FAP *plus* will be communicated directly to the treating staff within ~2 h of sampling.

**Discussion:**

We will examine if rapid use of FAP *plus* panel in hospitalized patients with suspected CAP can improve both the time to and the proportion of patients receiving pathogen-directed treatment, thereby shortening the exposure to unnecessary antibiotics and the length of hospital admission, compared to the standard-of-care arm. The pragmatic design together with broad inclusion criteria and a straightforward intervention could make our results generalizable to other similar centres.

**Trial registration:**

ClinicalTrials.govNCT04660084. Registered on December 9, 2020

**Supplementary Information:**

The online version contains supplementary material available at 10.1186/s13063-022-06467-7.

## Administrative information

Note: the numbers in curly brackets in this protocol refer to SPIRIT checklist item numbers. The order of the items has been modified to group similar items (see http://www.equator-network.org/reporting-guidelines/spirit-2013-statement-defining-standard-protocol-items-for-clinical-trials/).

**Table:** 

**Title {1}**	**Impact of Rapid Molecular Testing on Diagnosis, Treatment and Management of Community Acquired Pneumonia in Norway: a pragmatic randomised controlled trial (CAPNOR)**
Trial registration {2a and 2b}.	ClinicalTrials.gov: identifier number: NCT04660084 A pdf-file with the World Health Organization Trial Registration Data Set is shown in “Additional file 1”.
Protocol version {3}	Version 3, 21^st^ August 2020
Funding {4}	The Research Council of Norway (NORCAP; 288718) is the primary funder of the trial. Additional funding support is obtained from the Trond Mohn Foundation, the University of Bergen (UiB), and Haukeland University Hospital (HUH).
Author details {5a}	Sondre Serigstad^1,2^, Christian Ritz^3,4,^*, Daniel Faurholt-Jepsen^5^, Dagfinn Markussen^1^, Marit H. Ebbesen^6^, Øyvind Kommedal^3,6^, Rune O. Bjørneklett^1,2^, Lars Heggelund^3,7^, Tristan W. Clark^8^, Cornelis H. van Werkhoven^9^, Siri T. Knoop^6^, Elling Ulvestad^3,6^, Harleen M.S. Grewal^3,6,^*, and the CAPNOR study group *joint corresponding authors ^1^ Emergency Care Clinic, Haukeland University Hospital, Bergen, Norway ^2^ Department of Clinical Medicine, Faculty of Medicine, University of Bergen, Bergen, Norway ^3^ Department of Clinical Science, Bergen Integrated Diagnostic Stewardship cluster, Faculty of Medicine, University of Bergen, Bergen, Norway ^4^ National Institute of Public Health, University of Southern Denmark, Copenhagen, Denmark ^5^ Department of Infectious Diseases, Rigshospitalet, Copenhagen, Denmark. ^6^ Department of Microbiology, Haukeland University Hospital, Bergen, Norway ^7^ Department of Internal Medicine, Vestre Viken Hospital Trust, Drammen, Norway. ^8^ School of Clinical and Experimental Sciences, Faculty of Medicine, University of Southampton, Southampton, UK ^9^ Julius Center for Health Sciences and Primary Care, University Medical Center Utrecht, Utrecht, The Netherlands.
Name and contact information for the trial sponsor {5b}	University of Bergen Contact information: University of Bergen ​Jonas Lies veg 87 N-5021 Bergen
Role of sponsor {5c}	The sponsor and funders had no role in the study design, and they will have no role in the collection, management, analysis, and interpretation of data nor in the decision to submit the report for publication.

## Introduction

### Background and rationale {6a}

Lower respiratory tract infections, including community-acquired pneumonia (CAP), are a leading cause of hospital admissions and mortality in all age groups [1–5]. Pneumonia places a large burden on healthcare resources, with associated enormous annual costs in Europe, mainly due to hospitalization and absence from work [6].

Pneumonia is an infection of the lungs, with accompanying inflammation. Typical clinical symptoms include cough with or without sputum, fever, dyspnoea and chest pain [7]. However, elderly patients, who represent most admissions for pneumonia, present without many of these features. Moreover, chest radiography is hampered by suboptimal sensitivity to detect pulmonary infiltrates, especially in the initial phase of the disease and in elderly patients [8–10]. In light of the diagnostic difficulties, the reported incidence of hospital-treated CAP varies considerably: between 10 and 420 cases per 10,000 adults per year in developed countries, with higher numbers in children and elderly [11–13]. Similarly, estimated mortality rates are between 1 and 48% and consistently found to increase with age [5, 11]. A prospective study conducted at Drammen Hospital, Norway, found that 1- and 5-year mortality rates were 8.9% and 27.1%, respectively, among hospitalized CAP patients [14].

As CAP is a potentially serious infection, antibiotics are often given rapidly after presentation to hospital. Due to uncertain microbial aetiology, patients are initially treated with empirical antibiotics that cover a broad range of potential pathogens. The microbiological aetiology of CAP based on conventional routine microbiological testing is achieved in up to 30–40% of cases [12, 15]. With a few exceptions, standard-of-care diagnostic methods are too slow to influence early decisions on antimicrobial therapy. Culture-based techniques are also affected by prior use of antibiotics. Furthermore, sampling from the lower respiratory tract is problematic due to contamination and colonization from the upper respiratory tract flora. The management of CAP therefore largely relies on national empirical treatment guidelines that reflect the prevailing local epidemiology and microbial resistance profiles. Improving microbiological identification and enhancing rapid detection of significant pathogens could have a major impact on the decision-making process, by providing real-time information for treatment decisions within the emergency department. Rapid molecular testing for CAP aetiology may facilitate pathogen-directed treatment, reduce unnecessary antibiotic use, shorten length of hospital stay, improve viral detections and treatment and rationalize isolation facility use. However, limited evidence exists to support its use over standard-of-care microbiological testing. A recent randomised controlled trial (RCT) evaluated the use of molecular testing for respiratory viruses in hospitalized CAP patients in UK [16]. Molecular testing was associated with a greater proportion of patients being treated with brief courses of antibiotics, improved antiviral use, reduced length of stay and was considered safe. Moreover, respiratory viruses were detectable in up to 45% of hospitalized adults with acute respiratory illness [16]. These findings need confirming in other studies that include rapid molecular tests, targeting both viral and bacterial pathogens and with alternate outcome measures.

Tests using multiplex real-time polymerase chain reaction (PCR) can reduce the time to results compared with standard PCR panels from 12 to 48 h to about 2 h, with the potential to direct initial antimicrobial choice. The new generation BioFire® FilmArray® Pneumonia Panel *plus* (FAP *plus*) (bioMérieux S.A., Marcy-l’Etoile, France) includes the automated detection of 27 relevant bacterial (including atypical bacteria) and viral respiratory pathogens together with seven antimicrobial resistance genes [17]. The assay requires a 2-min hands-on time and a run time of about 1 h. We hypothesize that utilizing the FAP *plus* panel in CAP patients can improve both the time to and the proportion of patients receiving pathogen-directed treatment, thereby shortening the exposure to unnecessary antibiotics and the length of hospital admission.

### Hypotheses {7}

CAPNOR will assess the impact of a rapid molecular diagnostic approach (FAP *plus*) in adult CAP patients presenting to the emergency department, compared to the current standard-of-care microbiological testing. We hypothesize that the FAP *plus* may lead to an increase in the proportion of patients treated with, and shorten the time to, pathogen-directed treatment of CAP and so will reduce unnecessary use of broad-spectrum antibiotics for treatment of CAP.

### Trial design {8}

CAPNOR is a single-centre, single-blind, parallel-arm randomised controlled superiority trial. In an allocation ratio of 1:1, eligible patients will be randomised to receive either standard-of-care methods (standard microbiological testing) or standard-of-care methods in addition to testing by the FAP *plus*. The trial is pragmatic with a PRECIS-2 rating of the nine domains as follows: eligibility: 4, recruitment: 5, setting: 5, organization: 5, flexibility (delivery): 4, flexibility (adherence): 5, follow-up: 5, primary outcome: 5, primary analysis: 5 [18].

### Feasibility

To inform the trial, a prospective cohort feasibility study was carried out with 104 patients with suspected CAP [19]. The feasibility study mirrored the procedures described in the full-scale trial but without randomised of respiratory samples into two treatment arms. All lower respiratory tract samples were tested both by standard microbiological testing and by the FAP *plus*. The feasibility study was used to assess several parameters including the feasibility of recruitment, the included clinical and laboratory procedures and the usability and efficiency of the data entry process, data transfer and data analysis using the study-specific electronic case report form (eCRF) provided by Viedoc (Viedoc Technologies, Uppsala, Sweden). Thus, the feasibility study informed on any procedural or design refinements that were needed for the ensuing full-scale trial. Results from the feasibility study are reported elsewhere [19].

## Methods: participants, interventions and outcomes

### Study setting {9}

Over the 3-year period 2020–2023, CAP patients admitted to Haukeland University Hospital in Bergen, Western Norway, will be screened for inclusion into the CAPNOR study. HUH is a large academic hospital that serves as a local hospital for a population of approximately 470,000 persons and functions as a referral hospital for approximately 1,000,000 inhabitants. At admission, eligible patients with a suspicion of CAP will be recruited in the emergency department at HUH.

### Eligibility criteria {10}

#### Inclusion criteria

Adults (aged ≥18 years) presenting to the emergency department with a suspicion of CAP and fulfilling at least two of the following criteria: new or worsening cough, new or worsening expectoration of sputum, new or worsening dyspnoea, hemoptysis, pleuritic chest pain, radiological evidence of pneumonia, abnormalities on chest auscultation and/or percussion, and fever (≥38.0°C).

Written informed consent is needed from the patient or from their legal guardian/close relative at the time of recruitment.

#### Exclusion criteria

Any of the following conditions prohibit participation in the trial:

- Severe bronchiectasis (defined as patients in need of regular follow-up and treatment by a pulmonologist due to bronchiectasis)
- Cystic fibrosis
- A palliative approach (defined as life expectancy below 2 weeks)
- Hospitalization within the last 14 days prior to admission
- Patients not willing or able to provide a lower respiratory tract sample at admission

### Who will take informed consent? {26a}

The study physicians or study nurses will brief patients or the patient’s legally authorized representative, with regard to the nature of the study. Patients will be informed that their participation is voluntary and will receive information sheets. Patients or their legally authorized representative(s) will be required to sign a statement in an informed consent form (ICF) that meets the requirements of the Regional Committee for Medical and Health Research Ethics in Norway (REC), and this will be documented in the study eCRF. A copy of the ICF will be provided to the patient or the patient’s legally authorized representative. The ICF will contain a separate section that addresses the use of remaining mandatory samples for exploratory research. Patients are informed that they are free to refuse to participate and may withdraw their consent at any time and for any reason during the storage period of clinical samples.

### Additional consent provisions for collection and use of participant data and biological specimens {26b}

Patients are informed about the storage and use of their data and biological samples for future research on CAP. Informed consent is procured prior to collection of participant data and biological specimens. All biological specimens will be destroyed 5 years after the project ends.

## Interventions

### Explanation for the choice of comparators {6b}

The trial compares two types of microbiological testing approaches for the assessment of microbiological aetiology in CAP patients. The impact of novel rapid testing by the FAP *plus* is evaluated against standard microbiological testing (standard-of-care), which is the comparator arm.

### Intervention description {11a}

The trial evaluates the utility of diagnostic tests used for diagnosing the microbiological aetiology in CAP. Blood tests and cultures, urine sample for urine antigen tests, chest x-ray and samples from the respiratory tract are collected in all patients following inclusion. Due to local infection control measures, the patients’ SARS-CoV-2 status needs to be clarified before collecting samples from the lower respiratory tract. Following consent, all patients are tested for their SARS-CoV-2 status. A confirmed case of SARS-CoV-2 is defined as a positive result on the rapid XpertXpress SARS-CoV-2 or SARS-CoV-2/FLU/RSV test run on the GeneXpert system (Cepheid, Sunnyvale, U.A.) or on Cobas SARS- CoV-2 & Influenza A/B test run by the Cobas Liat System (Roche Molecular Systems, Inc., Pleasanton, CA) using naso-/oropharyngeal swab samples. Lower respiratory tract samples are collected after sputum induction or by endotracheal aspiration in both arms. On receipt of the lower respiratory tract sample at the Department of Microbiology, the patients are randomised to the following arms:

#### The standard-of-care arm

The comparator arm corresponds to standard-of-care diagnostics currently provided to CAP patients at HUH. As described [19], this includes bacterial culture of respiratory tract samples and blood according to current guidelines (adapted from [20]). Blood culture isolates and relevant respiratory isolates are identified with matrix-assisted laser desorption/ionization time-of-flight mass spectrometry (MALDI-ToF MS) using the Bruker’s microflex LT instrument, MBT Compass software ver. 4.1 and Compass Library DB-8468 (Bruker Daltonics, MA, USA). Nasopharyngeal and/or oropharyngeal swabs are examined by an in-house real-time PCR test to detect respiratory viruses and atypical bacteria (influenza A and B, human parainfluenza viruses 1-3, respiratory syncytial virus, human metapneumovirus, rhinovirus, SARS-CoV-2, *Bordetella pertussis*, *Bordetella parapertussis*, *Mycoplasma pneumoniae* and *Chlamydia pneumoniae*). The total turn-around time for the in-house PCR test is up to 48 h and is comparable to other centres nationally. Standard methods also include the pneumococcal urine antigen test (Quidel Corporation, San Diego, USA). Any additional tests requested by the treating physician are noted and counted as part of standard methods. A positive SARS-CoV-2 result, growth in blood cultures and positive pneumococcal antigen test results are phoned to the treating staff.

#### *The FAP* plus *arm*

The FAP *plus* arm entails extended and rapid diagnostics on lower respiratory tract samples and telephonic feedback to treating staff with results. The feedback does not involve advice on treatment. All patients will receive the standard microbiological tests described above, with additional testing by the FAP *plus*. Through a feedback loop, both negative and positive FAP *plus* results are telephonically communicated to the treating staff.

### Criteria for discontinuing or modifying allocated interventions {11b}

A patient can withdraw from the study at any time at his/her own request. If the patient withdraws consent, the investigators may retain and continue to use any data collected and analysed before such a withdrawal of consent. If a patient withdraws from the study, samples collected and not tested will be destroyed and the investigator will document this in the study records.

### Strategies to improve adherence to interventions {11c}

Not applicable as the two interventions do not require patients to participate actively.

### Relevant concomitant care permitted or prohibited during the trial {11d}

Any additional microbiological methods introduced by the treating physicians are counted within the repertoire of standard diagnostic methods provided.

### Provisions for post-trial care {30}

Not applicable. The interventions do not involve active involvement or participation from the patients beyond providing informed consent as the trial is evaluating the utility of diagnostic tests used for diagnosing the aetiology in CAP, i.e., medical tests for determining the aetiology in CAP, and not treatments of CAP, are compared.

### Outcomes {12}

#### Primary outcomes

There are two primary outcome variables:

1. Provision of pathogen-directed treatment based on a microbiological test result deemed as clinically relevant within 48 h of receipt of respiratory samples. This is a binary outcome variable taking on values as follows: yes, if such treatment was given to the patient, and no, if it was not given.
2. Time (in hours) from receipt of respiratory samples to the patient receiving pathogen-directed treatment. This is a quantitative outcome variable recording the time elapsed from receipt of respiratory samples to provision of pathogen-directed treatment based on a microbiological test result deemed as clinically relevant (as defined in the first primary outcome) or an elapse of 48 h, whichever event came first. In other words, this outcome variable is subject to right censoring at 48 h. Right censoring could potentially occur for other reasons such as no aetiology being detected or the patient dying.

Since lack of microbiological identification is a major problem in CAP patients and enhancing rapid detection of significant pathogens could have a major impact on the clinical decision-making process, we selected two correlated primary outcomes with both time to and provision of pathogen-directed treatment. Limited evidence exists to support the use of these new molecular microbiological methods over standard of care, which uses standard microbiological testing. In a retrospective study including rapid molecular diagnostics, it has been suggested that de-escalation from broad-spectrum to pathogen-directed antibiotics could be undertaken in 77% of patients with CAP, while escalation should be done in 6%; however, a RCT is needed to confirm these findings [21].

#### Secondary outcomes

There are 13 binary outcomes (encoded as yes/no):

1. Treatment with narrow-spectrum antibiotics within 48 h from study inclusion
2. Treatment with a single dose of antibiotics only
3. Treatment with antibiotics for not more than 48 h (within the first 7 days after inclusion)
4. Treatment with intravenous antibiotics (within the first 7 days after inclusion)
5. De-escalation from broad-spectrum to narrow-spectrum antibiotics (within the first 7 days after inclusion)
6. Escalation from narrow-spectrum to broad-spectrum antibiotics (within the first 7 days after inclusion)
7. Detected aetiology of CAP (within the first 7 days after inclusion)
8. Provision of neuraminidase inhibitors to patients with diagnosed influenza (within the first 7 days after inclusion)
9. Readmission within 30 days after discharge
10. Death within 30 days, 90 days, 1 year and 5 years

There are eight quantitative outcomes:

1. Duration of antibiotic use; intravenous and per-oral (in days)
2. Duration of intravenous antibiotics (in days)
3. Duration of broad-spectrum antibiotics (in days)
4. Time from admission to time of pathogen-directed treatment (in hours)
5. Time from admission to time of administration of antibiotic(s) (in hours)
6. Time to appropriate use of isolation facilities (in days)
7. Length of hospital stay (in days)
8. Time from admission to a microbiological sputum test report (FAP *plus* result and/or sputum culture) (in hours)

#### Exploratory outcomes

- Explore host-derived diagnostic markers and markers that predict the clinical outcome by protein and transcriptional profiling assays (collected at defined time points (Fig. 2) during the hospital stay)
- Compare evaluation of sputum quality when judged by traditional microscopic criteria (performed as part of standard of care by clinical microbiologists at the laboratory) versus pre-specified macroscopic criteria for manual inspection (by the sputum collecting personnel in the emergency department)
- Explore the aetiology and hospital management of CAP in relation to earlier exposure to antibiotics, vaccination, comorbidity, medication, smoking status, alcohol use, travelling history, hospitalization and frailty indicators including nursing home residency and scoring systems (clinical frailty scale)

Furthermore, several additional outcomes will be used to explore timings of the individual parts of the interventions:

A. Time (in minutes) used for obtaining a lower respiratory tract sample (time from start to stop of the induced sputum/endotracheal aspiration procedure)
B. Time used from admission to time of:

- Inclusion (in minutes)
- Obtaining induced sputum/tracheal aspirate (in minutes)
- Sampling throat swab for SARS-CoV-2 (in minutes)
- Randomised (in minutes)
- A microbiological test result obtained from the sputum sample (time of the sputum culture report and/or time of the FAP *plus* result) (in hours)

### Participant timeline {13}

**Fig. 1:**
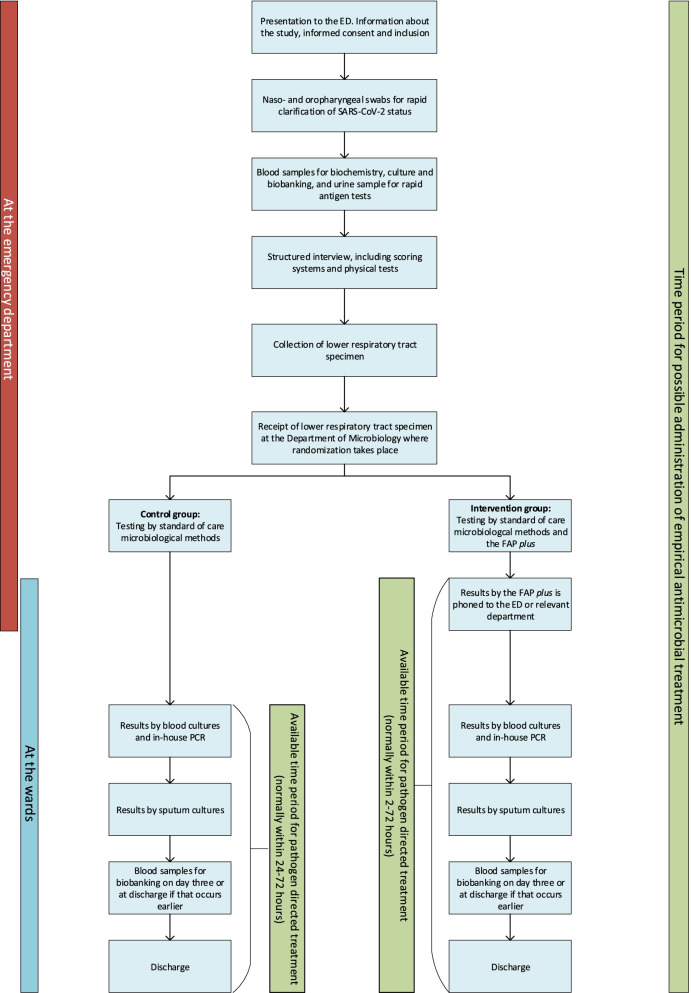
Participant timeline. Overview of the participant timeline. *Abbreviations:* ED, emergency department; FAP *plus*, BioFire® FilmArray® Pneumonia Panel *plus* (bioMérieux S.A., Marcy-l’Etoile, France); PCR, polymerase chain reaction

**Fig. 2:**
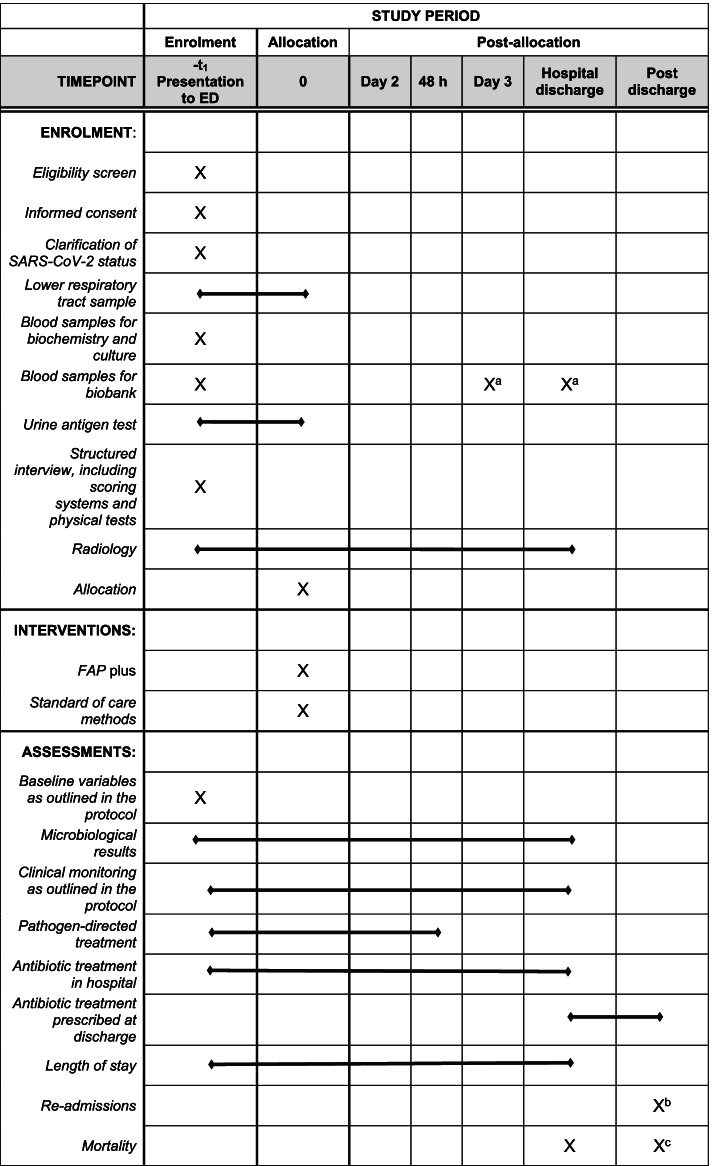
Schedule of enrolment, interventions and assessments. ^a^At discharge or on day 3, whichever comes first. ^b^Day 30. ^c^Post-discharge at day 30, day 90, 1 year and 5 years. *Abbreviations:* ED, emergency department; FAP *plus*, BioFire® FilmArray® Pneumonia Panel *plus* (bioMérieux S.A., Marcy-l’Etoile, France)

The participant timeline is shown in Fig. 1. A schematic diagram with the time schedules for enrolment, interventions and assessments for participants is shown in Fig. 2. Eligible patients are identified and included in the emergency department. Due to local infection control regulations, the patient’s SARS-CoV-2 status is clarified before collection of lower respiratory tract samples. Following consent, all suspected CAP patients are tested for SARS-CoV-2 by naso- and oropharyngeal swab samples. In both SARS-CoV-2- negative and SARS-CoV-2-positive patients, collection of lower respiratory tract samples is performed as soon as possible in the emergency department, either by induced sputum or by endotracheal aspiration. Spontaneous sputum is collected as an exception if an induced sputum cannot be collected. If sputum induction is unsuccessful, endotracheal aspiration is performed. Respiratory tract samples are sent immediately via a pipeline system to the Department of Microbiology at HUH, where microbiological testing takes place. In patients randomised to the FAP *plus*, the test result is phoned to the treating staff.

Blood samples for standard biochemistry and cultures, urine for antigen testing (*Streptococcus pneumoniae*) and clinical data collection including scoring systems and physical tests are performed at the emergency department. Furthermore, at admission and on day 3, blood ethylenediamine tetra-acetic acid- (EDTA) and PAXgene blood RNA tubes (PreAnalytiX, Hombrechtikon, Switzerland) are collected and subsequently frozen in a biobank at HUH.

### Sample size {14}

To ensure a sufficiently large sample size to address the multiplicity issue introduced by considering two primary outcomes, we take a slightly conservative approach of assuming that the two outcomes are uncorrelated, implying that separate sample size calculations for the two outcomes must be carried out at a significance level of 0.025 instead of 0.05.

In a prospective study on hospitalized CAP patients, with stringent inclusion criteria (that included the presence of a new pulmonary infiltrate on chest radiograph), we showed that microbial aetiology with a combination of molecular and conventional methods could be established in 63% of included patients [22]. Therefore, for the proportion of patients with change in treatment from empirical antimicrobials to pathogen-guided treatment, we expect to be able to identify a pathogen in at least 50% of the cases using FAP *plus* versus 40% with the current standard-of-care methods.

The required sample size needed is 470 per arm (assuming a significance level of 0.025 and a power of 0.8), i.e., in total 940. Additionally, allowing for a 10% dropout rate results in a total sample size of 1045 patients. For the time to change from empirical treatment to pathogen-directed treatment, we have no data from previous studies on effect size to gauge the sample size calculation and therefore we will define the effect size in terms of the variation in the outcome (the standard deviation) as done in other studies [23].

We find it clinically relevant to be able to detect a difference of 0.2 standard deviations, e.g., if the standard deviation of the time to change is 3 days, then we can detect a difference of 0.2‧3 = 0.6 day; if the standard deviation was 5 days, we would detect a difference of 1 day. The required sample size needed is 477 per arm (assuming a significance level of 0.025 and a power of 0.8), i.e., in total 954. Additionally, allowing for a 10% dropout rate results in a total sample size of 1060 patients.

In conclusion, a sample size of 1060 will ensure that we have 80% power to detect a difference in at least one of the two primary outcomes (at a significance level of 0.05).

### Recruitment {15}

The number of CAP patients admitted to Bergen Hospital Trust, including the emergency department at HUH, from March 2020 to March 2021 was approximately 1200 [24]. By including suspected CAP patients Monday until Friday between 8 am and 9 pm, we expect to be able to recruit ~350 CAP patients per year. Therefore, enrolment is expected to be completed within the 3-year period: 2020–2023.

## Assignment of interventions: allocation

### Sequence generation {16a}

The allocation sequence is computer-generated using the extension package “blockrand” within the statistical environment R (version 3.6.3; Vienna, Austria) with a pre-specified seed number for initiating the random number generator in R. Specifically, blocked randomised is applied with blocks with 4, 6 or 8 patients; the block sizes will occur in random order to ensure approximately equal allocation over the year.

### Concealment mechanism {16b}

The generated allocation sequence is prepared and entered in the eCRF (Viedoc) by staff not involved in the trial. It remains concealed until the moment the patient is randomised, which happens once the Department of Microbiology at HUH receives the respiratory tract sample.

### Implementation {16c}

The patients are included at the emergency department by study nurses and study physicians. When the Department of Microbiology receives the respiratory tract specimen, a bioengineer initiates the randomised process that is implemented in Viedoc. The result of the allocation is made available for the staff at the Department of Microbiology in the laboratory’s electronic data system and guides the processing of the lower respiratory tract samples into one of the arms.

### Assignment of interventions: blinding

### Who will be blinded {17a}

CAP patients are blinded for the intervention, and medical staff in the emergency department are blinded at patient inclusion and hence have no influence on allocation which is assigned at the Department of Microbiology, HUH. Owing to the nature of the intervention, research staff and clinical care providers are not blinded to group allocation. Furthermore, data managers, outcome assessors and statisticians are not blinded. However, in contrast to data managers and outcome assessors, the involved statisticians do not have any direct access to data, which are stored within the Viedoc platform for patient registry at HUH.

### Procedure for unblinding if needed {17b}

Not applicable. Our intervention in the FAP *plus* arm involves microbiological diagnostic testing only. Furthermore, the allocation will be visible in the patient case record in the intervention group when the result of the FAP *plus* are available, normally within 1–2 h.

## Data collection and management

### Plans for assessment and collection of outcomes {18a}

Baseline information is collected by study nurses or investigating physicians through a structured interview in the emergency department. Symptoms and findings upon clinical examinations are recorded. Data pertaining to antimicrobial treatment and decisions, results from laboratory tests and medical imaging are obtained from electronic medical records and charts, after patient discharge, and used for the evaluation of the primary outcomes. For each patient, two experienced physicians separately evaluate the clinical relevance of all microbiological findings [19] and determine if and when the patient received pathogen-directed antimicrobial treatment based on a microbiological test result. In case of any inconsistency between the two physicians, a third independent physician will arbitrate. To be considered as pathogen-directed treatment, study physicians will note if (a) there is a change in antimicrobial treatment based on a microbiology test result or (b) a continuation of already correctly initiated antimicrobial treatment based on a microbiology test result, and (c) discontinuation of antimicrobial treatment based on negative microbiological test result(s). Empirical and pathogen-directed therapy will be determined using national guidelines recommended by the Norwegian Directorate of Health, data from national susceptibility reports, as well as results from anti-microbial susceptibility testing provided by the Department of Microbiology, HUH, as appropriate [25, 26]. Data will be registered in our eCRF (Viedoc). Data for the 30- and 90-day mortality, and 1- and 5-year mortality will be obtained from the Norwegian Cause of Death Registry.

#### Microbiological sampling and methods

Microbiological sampling and methods are performed as described [19]:

At inclusion, a lower respiratory tract sample used for the FAP *plus* and standard culture is obtained from all patients as soon as possible in the emergency department, either by induced sputum or by endotracheal aspiration. Depending on clinical symptoms, vital signs and medical history, sputum is induced either by nebulized isotonic (0.9%) or hypertonic (5.8%) saline. Patients with known obstructive lung disease and patients with hypoxemia or signs of airway obstruction upon physical examination are additionally treated with a bronchodilator (salbutamol and/or ipratropium bromide) prior to sampling. If sputum induction is unsuccessful, endotracheal aspiration is performed. Spontaneous sputum is accepted as an exception if an induced sputum or endotracheal aspiration cannot be collected

The standard methods include culture of respiratory tract samples and blood according to current guidelines (adapted from [20]). Blood culture isolates and relevant respiratory isolates are identified with matrix-assisted laser desorption/ionization time-of-flight mass spectrometry (MALDI-ToF MS) using Bruker’s microflex LT instrument, MBT Compass software ver. 4.1 and Compass Library DB-8468 (Bruker Daltonics, MA, USA). Nasopharyngeal and/or oropharyngeal swabs will be examined by an in-house real-time PCR test to detect respiratory viruses and atypical bacteria (influenza A and B, human parainfluenza viruses 1-3, respiratory syncytial virus, human metapneumovirus, rhinovirus, SARS-CoV-2, *Bordetella pertussis*, *Bordetella parapertussis*, *Mycoplasma pneumoniae* and *Chlamydia pneumoniae*). Standard methods also include the pneumococcal urine antigen test (Quidel Corporation, San Diego, USA). Any additional tests requested by the treating physician are also noted and counted as part of standard methods.

The representativeness of all sputum samples is evaluated by Gram staining (adapted from 22). Samples containing ≥ 10 squamous epithelial cells per field in at least 10 fields with 10× enlargement are considered non-representative. However, this criterion is disregarded if a significant amount of both leukocytes (≥ 10 times the amount of squamous epithelial cells per field of view) and a morphologically uniform microbe (> 5 microbes per field of view with 100× enlargement) are present. Samples are analysed by the FAP *plus* and cultured on agar plates, irrespective of their representativeness. Abundant growth of plausible respiratory pathogens is reported regardless of the representativeness of the sputum sample. Non-abundant growth is only considered in samples considered representative.

The FAP *plus* is an automated multiplex PCR test validated for lower respiratory tract samples. It is capable to detect 27 bacteria and viruses, as well as seven genetic markers of antibiotic resistance. The hands-on time is around 2 min and the total analysis time about 1 h [17]. Bacterial detections (except the atypical bacteria) are reported in a semi-quantitative manner and categorized as negative if ≤10^3.5^ copies/ml. Above this level, results are reported as positive and semi-quantitatively specified as 10^4^, 10^5^, 10^6^ or ≥ 10^7^ copies/ml [27].

#### Scoring systems and nutritional and physical status

Anthropometry, clinical scoring systems and physical tests are performed at the emergency department. Height, weight and body mass index (BMI) are registered and calculated. Physical capacity (muscle strength) is measured with a handgrip test adapted from [28]. Risk stratification of patients at admission is required to guide management and treatment decisions. The most established score systems (such as the CURB-65 score, Pneumonia Severity Index (PSI) and Sequential Organ Failure Assessment (SOFA) score) may accurately predict the severity and mortality in some CAP patients, but do not automatically identify patients that benefit from aggressive management strategies. We will register and evaluate several validated scoring systems: the CRB-65-score, CURB-65-score, SOFA-score, PSI-score, Charlton Comorbidity Index, Clinical Frailty scale, South African Triage Scale (SATS), Triage Early Warning Score (TEWS) and National Early Warning score 1 and 2 (NEWS 1 and 2). In addition, we will explore potential cognitive impairment at admission using the Abbreviated Mental Test 4 (4AT-score). Test calculators and form are available online [29–31].

Micronutrients important to immune function and CAP such as 25-OH vitamin D will be measured. Specifically, vitamin D regulates the production of antimicrobial peptides (cathelicidin and beta-defensin-2), which play an important role in the innate immune response to infection [32]. Data on habitual alcohol consumption and smoking will also be collected. Diabetes status will be assessed using HbA1c [33]. All participants will be tested at admission to identify unknown/new-onset diabetes and pre-diabetes, to differentiate admission hyperglycaemia between patients with and without diabetes (exhibit different risk profiles). We will assess acute dysregulation (the glycaemic gap) by comparing admission plasma glucose to estimated mean plasma glucose derived from HbA1c [34]. The cut-off values for both fasting and postprandial hyperglycaemia among the patients without diabetes will be defined by receiver operator curve analysis. In addition, we will record any other pre-admission comorbidities, such as chronic obstructive lung disease, heart disease and kidney disease.

#### Health economic data

Health economic data will be computed by combining information on molecular test performances, linked to consequences for costs for diagnosis and treatment. The Department of Microbiology, HUH, has developed a well-evaluated model for estimating the total cost of performing tests that includes reagents, technician time, instrument cost etc. This model will be used to calculate the actual cost of performing the FAP *plus*. The data collected will be used to conduct exploratory economic analyses and will include number and duration of medical care encounters, duration of hospitalization (total days or length of stay), number and type of diagnostic and therapeutic tests and procedures, potential cost reductions related to reduced hospital stay, reduced use of isolation rooms and possibly fewer days admitted to the intensive care unit and other factors like reduction in the use of antibiotics and more rapid transfer to per oral treatment will also be included.

#### Biobank

The respiratory tract samples will be frozen and stored in the biobank at HUH. Additional blood for transcriptional and immune marker profiling of patients (performed retrospectively) will be taken at admission and on day 3 of admission.

#### Biomarkers

Quantification of multiple proteins in selected clinical samples will be assessed by the Multiplex Bead Array-Bio-Plex assay (Bio-Rad Laboratories, Inc., CA, USA) using custom-designed human chemokine/cytokine kits and measured by the Bio-Plex 200 System (Bio-Rad Laboratories, Inc., CA, USA) with Luminex xMAP technology. For transcriptional profiling, we will target gene panels that include T and B cell markers as well as type 1 interferon-inducible genes. In addition, classifier genes that discriminate between viral and bacterial aetiology will be evaluated.

### Plans to promote participant retention and complete follow-up {18b}

The participants are included in the emergency department and the sampling of microbiological samples is done at the same time. We therefore do not need to promote participant retention.

### Data management {19}

Data will be entered into standardized patient-specific eCRFs provided by Viedoc, a commercial electronic clinical data entry system, which is used at HUH. All clinical and microbiological data entries (including range checks) are double-checked by study staff. The Viedoc application uses redundant enterprise-level storage area networks to store all data on separate data centres at separate geographic locations. In addition to the doubled data storage and application servers, a backup of all data is taken once every 2 h. Every 24 h, a copy of the latest backup is transferred to a third separate geographic location, and once a week, a copy of the backups is stored in a bank vault.

### Confidentiality {27}

Relevant data is entered into our study-specific eCRF created in the generic eCRF provided by Viedoc. Only designated and authorized personnel that are part of the study have access to the database. Participants are assigned a unique identifier. Any participant records or datasets that are transferred will contain the identifier only; participant names or any information which would make the participant identifiable are not transferred. The participants are required to give consent for their data to be used as described in the informed consent. For patients randomised to the FAP *plus* test, the results are made available in the patients’ electronic medical journal. The Department of Microbiology at HUH ensures the integrity and confidentiality of all microbiological data of enrolled patients by providing unique identification (ID) numbers for all person-identifiable data. All patient samples are assigned a unique sample ID (generated by the laboratory information system Unilab-700 (Alphasoft GmbH, Bochum, Germany)). Samples from patients enrolled in the CAPNOR study will be further assigned a CAPNOR study ID. At HUH, only authorized physicians employed at the Department of Microbiology or at the Department of Infectious Diseases have access to the UNILAB ID and to patient identifiable data.

### Plans for collection, laboratory evaluation and storage of biological specimens for genetic or molecular analysis in this trial/future use {33}

The respiratory tract samples collected at admission are frozen after analysis and stored at the Biobank at HUH. Plasma samples and PAXgene tubes collected at admission and at day 3 are also frozen and stored in the Biobank. Plasma and PAXgene tubes will be used for transcriptional and immune marker profiling.

## Statistical methods

### Statistical methods for primary and secondary outcomes {20a}

For the first primary outcome, which is a binary outcome capturing whether or not provision of pathogen-directed treatment was given, the comparison between arms will be performed using a logistic regression model. The difference between arms will be quantified by means of an odds ratio or as a difference in probabilities (“a risk difference”). For the second primary outcome, which is a quantitative outcome subject to right censoring capturing the time to pathogen-directed treatment was given, the comparison between arms will be carried out using a semi-parametric or fully parametric event-time model such as the Cox proportional hazards model or accelerated failure time model. Relevant covariate adjustment, including age and sex, will be included in both models.

Secondary outcomes will be analysed using the same two types of models as used for the two primary outcomes. However, for some quantitative secondary outcomes, there will be no right censoring present and, in such cases, linear models or linear mixed models will be fitted, depending on the absence or presence of repeated measurements.

### Interim analyses {21b}

No interim analysis is planned.

### Methods for additional analyses (e.g. subgroup analyses) {20b}

Similar statistical models as detailed for the primary and secondary outcomes above will be applied but include interaction terms between the treatment variable and the variables defining the subgroups. Specifically, differences in outcomes (between the two treatment arms) will be investigated for the following subgroups:

- Pathogen-specific subgroups (bacterial, viral, combined bacterial and viral group and those with unknown aetiology individually)
- Radiologically confirmed CAP and clinically suspected CAP
- Patients with severe versus non-severe pneumonia, based on different scoring systems
- Sputum samples judged as representative versus those judged as not representative by microscopic criteria
- Hospital ward allocation
- Patients with chronic pulmonary disease (chronic obstructive pulmonary disease, asthma, bronchiectasis) versus those without
- Use of antibiotics: on admission, within the preceding month prior to admission, within 48 h prior to admission

### Methods in analysis to handle protocol non-adherence and any statistical methods to handle missing data {20c}

As CAPNOR is a pragmatic trial, the main statistical analyses will be carried out according to the intention-to-treat principle using appropriate effectiveness estimands [40]. Specifically, for the two primary outcomes, which are based on an event time, possibly subject to right censoring, the statistical analysis will be based on all patients that were randomised. Likewise, for event-time-derived secondary outcomes, statistical analyses will also be based on the full set of randomised patients. For other outcomes, where missing values may occur, missing values will be imputed using outcome-specific imputation models unless complete-case or available-case analyses could be justified because the missing data mechanisms are missing completely at random or missing at random, respectively.

### Plans to give access to the full protocol, participant-level data and statistical code {31c}

The full protocol and scripts used for the statistical analysis will be available on request once results of the study have been published. Patient-level data will not be available.

## Oversight and monitoring

### Composition of the coordinating centre and trial steering committee {5d}

The principal investigator (PI) will be scientifically responsible and responsible for communication internally within the consortium and with the main funder (Research Council of Norway). This includes compiling and submission of progress reports and financial reports. The PI will be supported by the core project management group (comprising senior CAPNOR group members based at the University of Bergen (UiB) and HUH), who will conduct regular meetings to monitor trial progress. In addition, advice will be sought on a case-to-case basis from the project’s independent scientific advisory committee. Responsibility for the data management and the contact person for questions regarding the use of research data lie with the project’s PI. The project’s data management team include three bioengineers (HUH), local Viedoc support designee, study nurses (three based at HUH), study doctors (four based at HUH/UIB) and a post-doctoral scientist (based at UIB).

### Composition of the data monitoring committee, its role and reporting structure {21a}

The study was reviewed by the sponsor and felt to be of low risk on the grounds that it is not a clinical trial of a medical treatment requiring active involvement or participation by patients. Therefore, the likelihood of harms associated with the interventions was judged to be low (see also {22} below), waiving the need for a data monitoring committee.

### Adverse event reporting and harms {22}

The FAP *plus* has demonstrated excellent sensitivity and specificity in multicentre evaluations [35, 36], and we consider the risks associated with this test to be low. Respiratory samples collected on admission are recommended as part of the standard of care, and our intervention therefore causes no additional risk. The risk of blood sampling in the form of pain, bleeding and infection is minimal. No other adverse events are anticipated, moreover, and no adverse effects were reported in our feasibility study [19]. However, monitoring and reporting of adverse events and severe adverse events will take place throughout the trial period. Any suspicion of trial-related adverse events will immediately be discussed in the CAPNOR study group, documented in our eCRF and reported, if applicable, in the trial publication. Treatment recommendations to escalate, de-escalate or stop antibiotic treatment may be beneficial for the individual patient by minimizing exposure to antibiotics and/or improving pathogen-specific targeted use of antibiotics. Final decisions will always be made by the treating physician taking into account all clinical and diagnostic information.

### Frequency and plans for auditing trial conduct {23}

Regular monitoring will be performed according to ICH GCP (International Conference on Harmonisation-Good Clinical Practice) by the sponsor, who will verify that the clinical trial is conducted in compliance with the protocol, GCP and applicable regulatory requirements.

### Plans for communicating important protocol amendments to relevant parties (e.g. trial participants, ethical committees) {25}

Amendments made to the study protocol after having obtained initial ethical approval will be (1) submitted to the REC for approval, (2) communicated to the funding agencies and (3) communicated in the main publications of the results of the CAPNOR trial.

### Dissemination plans {31a}

The results of this study will be published and presented at scientific meetings. The investigators will comply with the requirements for publication of study results. Authorship linked to publications ensuing from the trial will be determined based on substantive intellectual contributions. All contributions credited as authors will take responsibility and be accountable for what is published. Authorships will be based on the International Committee of Medical Journal Editors criteria. Currently, we have no plans of using professional writers. In addition, technical bulletins on methodological updates will be disseminated to appropriate partners. These publications will be restricted to the consortium unless agreed otherwise by all involved partners (for example, to make press releases). Importantly, we will publish the results of the proposed study in peer-reviewed international journals and relevant data will be made available in appropriate databases. Partners who identify a legitimate commercial interest may request a delay of no more than 60 days to allow for filing of patents, in which case the rules for protection of intellectual property rights, which will be laid out in the consortium agreement, will be followed. The standard rules on data protection will be followed, in which premature release of data is discouraged. However, on completion of data analysis and study closure, publication in the peer-reviewed literature will follow without unnecessary delays.

## Discussion

The COVID-19 pandemic spread to Norway during March 2020 and forced us to adapt to new routines and infection control measures deployed at hospitals and in the society at large. The planned start of the trial was therefore delayed with about 6 months. The time available to collect data and complete inclusions at the emergency department was initially restricted due to shortage of isolation facilities and a large inflow of patients. Initially, each patient is screened for SARS-CoV-2 before an induced sputum sample can be collected at the emergency department, with the subsequent risk of delaying the lower respiratory tract sampling. This is minimized by rapidly sending an oro- or nasopharyngeal swab for analysis by a rapid SARS-CoV-2 test, which provides results within 1 h.

With the national infection control measures, including social distancing, use of facemasks and home office solutions, the number of hospital admissions for lower respiratory tract infections at Bergen Health Trust fell during the first year of the pandemic [24]. This has resulted in slower inclusion rates than anticipated. Finally, COVID-19 and related quarantine among study staff has led to problems with optimal recruitment and timely pursuit of pre-defined study milestones due to absenteeism.

## Trial status

Protocol version 3; 21 August 2020. On September 25, 2020, the first patient was enrolled. As of February 2022, 354 patients have been enrolled. Recruitment of patients is ongoing and expected to be completed in May 2023. The investigators may initiate study-site closure at any time, provided there is reasonable cause and sufficient notice is given in advance of the intended termination. Reasons for the early stopping may include, but are not limited to, demonstrated efficacy, futility or inadequate recruitment of participants by the investigators. If the study is prematurely terminated, the investigators will promptly inform the REC and the contract research organizations of the reason for termination.

## Supplementary Information

**Additional file 1. **World Health Organization Trial Registration Data Set.

## Data Availability

The final trial dataset will be available to CAPNOR investigators upon a written request. The use of the dataset by CAPNOR investigators for dissemination and publication purposes is regulated by a publication agreement, wherein the publication committee would approve specific requests for use of the dataset.

## Abbreviations

CAP: Community-acquired pneumonia
HUH: Haukeland University Hospital
RCT: Randomised controlled trial
PCR: Polymerase chain reaction
FAP *plus*: BioFire FilmArray Pneumonia Panel plus
UiB: University of Bergen
eCRF: Electronic case report form
ICF: Informed consent form
REC: Regional Committee for Medical and Health Research Ethics
EDTA: Ethylenediamine tetra-acetic acid
BMI: Body mass index
CURB-65-score: Confusion, urea, respiratory rate, blood pressure, age ≥ 65 years
PSI: Pneumonia Severity Index
SOFA: Sequential Organ Failure Assessment
CRB-65-score: Confusion, respiratory rate, blood pressure, age ≥ 65 years
SATS: South African Triage Scale
TEWS: Triage Early Warning Score
NEWS: National Early Warning score
4AT-score: Abbreviated Mental Test 4
ID: Identification
PI: Principal investigator
ICH GCP: International Conference on Harmonisation-Good Clinical Practice
