# Supplementary material for: Impact of rapid molecular testing on diagnosis, treatment and management of community-acquired pneumonia in Norway: a pragmatic randomised controlled trial (CAPNOR)
Source: Trials. 2022 Aug 1;23:622. doi: 10.1186/s13063-022-06467-7 (PMC9340738; doi:10.1186/s13063-022-06467-7)
Supplement: Supplementary file 1 — Additional file 1. World Health Organization Trial Registration Data Set. [file 13063_2022_6467_MOESM1_ESM.pdf]

Additional file 1:

World Health Organization Trial Registration Data Set	
**Data category**	**Information**
Primary registry and trial identifying number	ClinicalTrials.gov<br>NCT04660084
Date of registration in primary registry	9th December, 2020
Secondary identifying numbers	HaukelandUH_31935
Source(s) of monetary or material support	The Research Council of Norway (NORCAP; 288718) is the primary funder of the trial. Additional funding support is obtained from the Trond Mohn Foundation, the University of Bergen (UiB), and Haukeland University Hospital (HUH).
Primary sponsor	University of Bergen
Secondary sponsor(s)	Haukeland University Hospital
Contact for public queries	Harleen M.S. Grewal, MD PhD<br>Email: Harleen.Grewal@uib.no
Contact for scientific queries	Harleen M.S. Grewal, MD PhD<br>Email: Harleen.Grewal@uib.no
Public title	Impact of Rapid Molecular Testing on Diagnosis, Treatment and Management of Community Acquired Pneumonia in Norway: a pragmatic randomised controlled trial
Scientific title	Impact of Rapid Molecular Testing on Diagnosis, Treatment and Management of Community Acquired Pneumonia in Norway: a pragmatic randomised controlled trial
Countries of recruitment	Norway
Health condition(s) or problem(s) studied	Respiratory tract infections, community acquired pneumonia, antibiotic treatment, rapid diagnostics
Intervention(s)	Intervention arm: Samples from the lower respiratory tract are analysed by the rapid and comprehensive real-time multiplex PCR panel, the BioFire® FilmArray® Pneumonia panel *plus* (FAP *plus*), in addition to standard of care microbiological methods.
	Comparator arm: Samples from the lower respiratory tract are analysed by standard of care microbiological methods.
Key inclusion and exclusion criteria	*Inclusion criteria*<br>Adults (aged ≥18 years) presenting to the emergency department with a suspicion of CAP and fulfilling at least two of the following criteria: new or worsening cough; new or worsening expectoration of sputum; new or worsening dyspnoea; haemoptysis; pleuritic chest pain; radiological evidence of pneumonia;

	abnormalities on chest auscultation and/or percussion; fever (≥38.0^{o}C).<br>Written informed consent is needed from the patient or from their legal guardian/close relative at the time of recruitment.
	Exclusion criteria<br>Any of the following conditions prohibit participation in the trial:<br>- Severe bronchiectasis (defined as patients in need of regular follow-up and treatment by a pulmonologist due to bronchiectasis)<br>- Cystic fibrosis<br>- A palliative approach (defined as life expectancy below two weeks)<br>- Hospitalization within the last 14 days prior to admission<br>- Patients not willing or able to provide a lower respiratory tract sample at admission
Study type	Interventional (Clinical Trial)
	A pragmatic, single-blind, single-centre randomised controlled trial
	Primary purpose: Diagnostic
Date of first enrolment	September 2020
Target sample size	1060
Recruitment status	Recruiting
Primary outcome(s)	There are two primary outcome variables:<br>**1)** Provision of pathogen-directed treatment based on a microbiological test result deemed as clinically relevant within 48 hours of receipt of respiratory samples. This is a binary outcome variable taking on values: yes, if such treatment was given to the patient and no, if it was not given.<br>**2)** Time (in hours) from receipt of respiratory samples to the patient receiving pathogen-directed treatment. This is a quantitative outcome variable recording the time elapsed from receipt of respiratory samples to provision of pathogen-directed treatment based on a microbiological test result deemed as clinically relevant (as defined in the first primary outcome) or an elapse of 48 hours, whichever event came first. In other words, this outcome variable is subject to right censoring at 48 hours. Right censoring could potentially occur for other reasons such as no aetiology being detected or the patient dying.

Key secondary outcomes	• Treatment with intravenous antibiotics (within the first seven days after inclusion) (yes/no)<br>• Treatment with narrow-spectrum antibiotics within 48 hours from study inclusion (yes/no)<br>• De-escalation from broad-spectrum to narrow-spectrum antibiotics (within the first seven days after inclusion) (yes/no)<br>• Escalation from narrow-spectrum to broad-spectrum antibiotics (within the first seven days after inclusion) (yes/no)<br>• Detected aetiology of CAP (within the first seven days after inclusion) (yes/no)<br>• Duration of antibiotic use; intravenous and per-oral (in days)<br>• Duration of intravenous antibiotics (in days)<br>• Duration of broad-spectrum antibiotics (in days)<br>• Length of hospital stay (in days)
Ethics review	The Regional Committee for Medical and Health Research Ethics in Southeast Norway approved the 3th version of the protocol on 21st August, 2020 (registration no.: 31935)
